# Influence of Dimethacrylate Monomer on the Polymerization Efficacy of Resin-Based Dental Cements—FTIR Analysis

**DOI:** 10.3390/polym14020247

**Published:** 2022-01-07

**Authors:** Aleksandra Maletin, Ivan Ristic, Tanja Veljovic, Bojana Ramic, Tatjana Puskar, Milica Jeremic-Knezevic, Daniela Djurovic Koprivica, Bojana Milekic, Karolina Vukoje

**Affiliations:** 1 Department of Dental Medicine, Faculty of Medicine, University of Novi Sad, 21000 Novi Sad, Serbia; tanja.veljovic@mf.uns.ac.rs (T.V.); bojana.ramic@mf.uns.ac.rs (B.R.); milica.jeremic-knezevic@mf.uns.ac.rs (M.J.-K.); daniela.djurovic-koprivica@mf.uns.ac.rs (D.D.K.); karolina.vukoje@mf.uns.ac.rs (K.V.); 2 Department of Materials Engineering, Faculty of Technology, University of Novi Sad, 21000 Novi Sad, Serbia; ivan.ristic@uns.ac.rs; 3 Dentistry Clinic of Vojvodina, Department of Dental Medicine, Faculty of Medicine, University of Novi Sad, 21000 Novi Sad, Serbia; tatjana.puskar@mf.uns.ac.rs (T.P.); bojana.milekic@mf.uns.ac.rs (B.M.)

**Keywords:** resin-based dental cement, dimethacrylate monomer, BisGMA, FTIR analysis, polymerization efficacy

## Abstract

The degree of polymerization for dimethacrylate resin-based materials (BisGMA, TEGDMA, UDMA, HEMA) ranges from 55 to 75%. Literature data indicate that polymerization efficacy depends, among other factors, on the type of methacrylate resin comprising the material. The aim of this study was to evaluate the polymerization efficacy of four dental cement materials characterized by different polymerization mechanisms using FTIR analysis. In the present study, the FTIR method was adopted to analyze the degree of polymerization efficacy of four resin-based dental cement materials, two of which were self-cured and two were dual-cured cements. The IR spectral analysis was performed 24 h after the polymerization of the cementitious material. RelyX ARC cement exhibits the lowest polymerization efficacy (61.3%), while that of Variolink II (85.8%) and Maxcem Elite is the highest (90.1%). Although the efficacy of self-cured cements appears to be superior, the difference is not statistically significant (*p* = 0.280). Polymerization efficacy largely depends on the chemical structure of the material in terms of the presence of a particular methacrylate resin and less on the polymerization mechanism itself, i.e., whether it is a self-cured or dually cured dental cement. Thus, in clinical practice, cementitious materials with a higher proportion of TEGDMA compared with BisGMA are recommended.

## 1. Introduction

**Figure 1:**
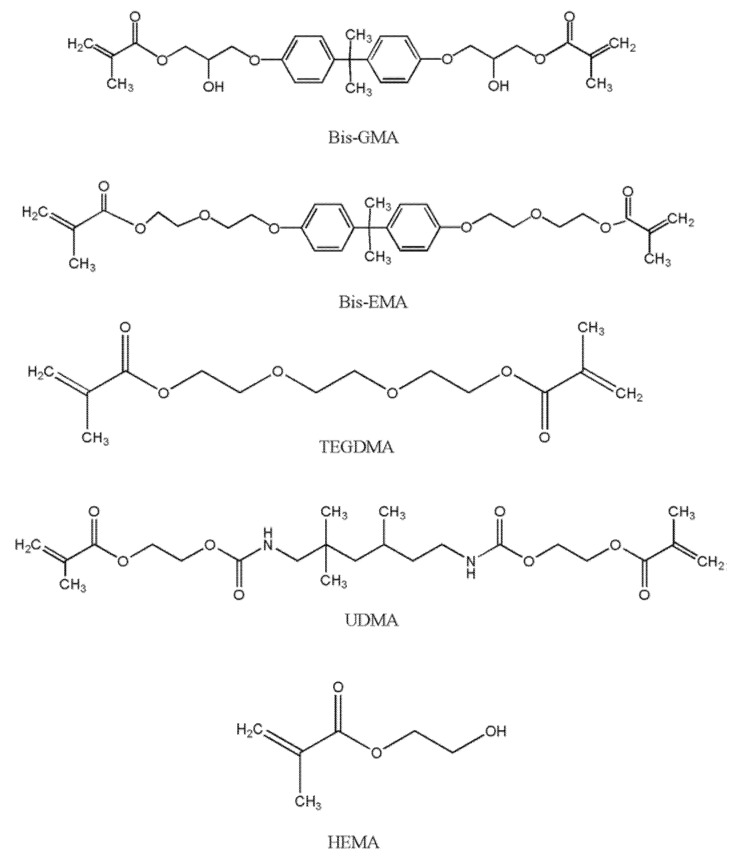
Dimethacrylate monomers.

In clinical practice, dental cement materials are primarily used for permanent and temporary fixation of indirect restorations to natural teeth [1,2]. According to the setting mechanism, dental cements are divided into water-based dental cements (zinc phosphate, zinc polycarboxylate and glass ionomer cements) and resin-based dental cements (composite cements) [3]. Resin-based dental cements exhibit superior mechanical, physical and adhesive properties compared with conventional cements. In contemporary dental practice, dental cements based on resins are successfully used for cementing highly aesthetic restorations, such as ceramic veneers, crowns and bridges, ceramic inlays and onlays and fiber posts [4]. Despite notable differences in their chemical composition, all composite materials comprise an organic resin matrix, inorganic filler and coupling agent (silane) [5,6]. Since 1962, when Bowen first introduced the bisphenol-A-glycidyl methacrylate (BisGMA) resin to the field of dentistry, composite materials have continued to develop [7]. The organic resin matrix is primarily constituted of dimethacrylate monomers such as BisGMA, bisphenol-A-ethoxy dimethacrylate (BisEMA) and/or urethane dimethacrylate (UDMA) (Figure 1) [8]. The presence of these molecules in cementitious materials results in outstanding mechanical properties, rapid polymerization and a low degree of polymerization contraction [9].

At room temperature, methyl methacrylate is a clear liquid of 0.945 g/mL density, with a molecular weight of 100 and a heat of polymerization of 129 kcal/mol. This exceptional reagent is typically polymerized chemically, whereby the polymerization reaction is induced by light and heat [5]. BisGMA belongs to the group of aromatic dimethacrylate esters and is synthesized from epoxy resin and methyl methacrylate [5]. BisGMA is a relatively rigid molecule with terminal methacrylate groups as the site of free radical polymerization and two benzene rings present near the center. The high degree of viscosity of BisGMA molecules is a consequence of the −OH group and hydrogen bonds. BisGMA is the most commonly used organic molecule in dental composite materials owing to its superior hardness and strength. The rigid aromatic structure of BisGMA is responsible for a lower degree of monomer-to-polymer conversion and higher modulus of elasticity, as well as a low degree of volatility and diffusion into oral tissues [10,11]. However, monomers of high molecular weight such as BisGMA increase the viscosity of dental composite materials, making their manipulation difficult, due to which some of the low molecular weight monomer diluents are also included in the structure, such as triethylene glycol dimethacrylate (TEGDMA), ethylene glycol dimethacrylate (EGDMA) and 2-hydroxyethyl methacrylate (HEMA) [8]. These diluents not only reduce the viscosity of BisGMA but also increase monomer crosslinking, thereby enhancing the polymerization efficacy [10].

The UDMA molecule in the form of a long linear chain contains one or more urethane groups and two methacrylate groups. Its molecular weight is comparable to that of BisGMA but it possesses greater flexibility and crosslinking capability [5]. Moreover, as UDMA can act as a hydrogen donor, its function is similar to that of the tertiary amine co-initiator, even though it is less effective in improving radical formation and polymerization rate. In addition, inclusion of UDMA into dental composite materials results in a stronger adhesive bond with the tooth structure [5].

HEMA is a low molecular weight fluid molecule that exhibits a very high allergenic potential in an uncured state. Due to its higher mobility and lower molecular weight, the unreacted monomer has the potential to diffuse into the tooth pulp and cause damage to the pulp cells. However, the presence of HEMA molecules is thought to increase the degree of polymerization efficacy because it has the ability to continue to react with unreacted carbon−carbon double bonds (C=C) even after most of the monomer crosslinking process has taken place [12].

TEGDMA is a diluent monomer characterized by low viscosity and a more flexible chain, resulting in reduced intermolecular bonding (due to the absence of –OH groups). Its use results in improved wettability and reduced hydrophobicity compared with BisGMA alone. Therefore, in clinical practice, BisGMA/TEGDMA co-monomer mixture is commonly used due to its high reactivity, which leads to a greater monomer mixture conversion rate. However, as the ether links present in both monomers increase the hydrophilicity of the cured system, this may ultimately result in higher water sorption and polymer degradation. The long-chain TEGDMA molecule is characterized by a relatively high degree of polymerization efficacy which, along with the consequent polymerization contraction, increases with TEGDMA percentage. However, the inclusion of TEGDMA compromises the beneficial mechanical characteristics of the dental material [10].

Fillers, as an inorganic structural component of resin-based cements, enhance compression and tensile strength, as well as modulus of elasticity [13]. Contraction stress and modulus of elasticity are directly proportional to the filler content in the resin-based cementitious material. The most common fillers are quartz, barium silicate, strontium silicate, zinc silicate, lithium aluminum silicate and yttrium and ytterbium trifluoride [14]. The establishment of a permanent bond between the filler and the organic resin matrix is enabled by the inclusion of a coupling agent into the composite material composition. The coupling agent is a bifunctional molecule, typically gamma-methaxyloxypropyltrimethoxy silane (γ-MPS), capable of establishing a bond with the hydroxyl groups of inorganic filler particles with the methacrylic groups of the organic resin matrix [15].

Polymerization occurs when monomers react to convert into polymers. The monomers used in dentistry are in liquid form and solidify as a result of polymerization, which has three phases denoted as initiation, propagation and termination. Free radicals are necessary to lengthen the polymer chain and are formed by photoinitiators. Hence, dental composites can be light-cured, self-cured or dually cured [5].

Chemical activation is a reaction between an organic amine-catalyst paste with an organic peroxide-universal paste. The mixing of these two pastes results in free radical formation. These free radicals attack the carbon double bonds, initiating a rapid polymerization process. While self-curable resins have similar composition to their light-cured counterparts, polymerization is initiated differently. For this purpose, benzoyl peroxide serves as the initiator and is frequently combined with an aromatic tertiary amine. By mixing the two pastes, amines react with benzoyl peroxide to form free radicals and initiate an addition-type polymerization reaction [13]. The most commonly used tertiary amines are N, N-dimethyl-p-toluidine and N, N-dihydroxyethyl-p-toluidine [16].

Photopolymerization requires light energy to initiate a photochemical reaction in a monomer. Adequate photopolymerization is critical for the optimal mechanical performance, biocompatibility and color stability of light-cured cements. Camphorquinone (CQ), with an absorption spectrum in the 390–510 nm range and an absorption maximum at 468 nm, is the most commonly used photoinitiator in the composition of light-cured cements [17].Tertiary amine is also included as a co-initiator into light-cured cements as it reacts with the activated photoinitiator to form free radicals. As camphorquinone gives an undesirable yellow color to the material, its concentration is limited, which has an adverse effect on polymerization efficacy. Consequently, it can be replaced by new photoinitiator systems such as 1-phenyl1,2-propanedione (PPD) and octyloxy-phenyl-iodonium hexafluoroantimonate (OPPI) to improve both the polymerization kinetics and the esthetics of the polymerized material. Although light-cured cements are easy to handle due to their controlled setting time, their structure only permits photopolymerization [18].

Dually cured dental materials address this shortcoming, as they rely on both light and self-polymerization. In these materials, polymerization is initiated by light exposure. They include photoinitiators such as CQ, iodonium salts and electron donors, which generate the reactive cationic species that start the polymerization process. When using dually cured materials, the polymerization reaction should be initiated with 10 s irradiation, after which 5 min are usually sufficient for the chemical part of the process. The use of dually cured cements in restorative dentistry is indicated when light polymerization may not be sufficient for adequate monomer conversion, which is typically the case when cementing fiber posts in the root canal [6].

The polymerization process is initiated by the free radicals that are generated during photoinitiation, which converts C=C bonds into C−C bonds between the generated radical and methacrylate group of the monomer molecule whereby the radical and alkene group of methacrylate donates an electron. The remaining electron of the alkene group reaches the opposite terminal of the monomer, due to which the whole molecule becomes a radical capable of reacting with another monomer. This process results in a chain reaction that terminates when two radicals react with one other. As the aim is to convert uncured resin into cured/polymerized resin, Degree of Conversion (DC) is used to select the most optimal resin [18].

Theoretically, during the polymerization process, all monomer molecules should be converted to polymers. However, dimethacrylate monomers exhibit a certain percentage of residual unreacted double C=C bonds in the polymer, resulting in a polymerization efficacy of 55–75% [10]. In clinical practice, polymerization efficacy is influenced by the physical and biological features of dental resin. Its value needs to be sufficiently high to prevent leakage of the unlinked molecules into the surrounding tissues.

The polymerization efficacy (which is never 100%) affects the mechanical characteristics and chemical stability of resin-based cements [19,20]. A lower polymerization efficacy can result in altered biomechanical properties in terms of reduced material hardness, increased hydrolytic degradation and diminished resistance to fracture and wear, as well as significant release of residual monomer, with adverse effects on the material biocompatibility [19,21]. Therefore, when the polymerization efficacy is suboptimal, the bond strength between the material and the tooth structure is inadequate [19]. For composite materials currently available on the dental market, polymerization efficacy varies between 36 and 67% [18].

The degree of polymerization efficacy is determined by applying various methods, such as micro Raman spectroscopy, Fourier transform infrared spectroscopy (FTIR), differential thermal analysis (DTA) and differential scanning calorimetry (DSC) [7,22,23]. DSC is typically employed to measure the heat generated during a polymerization reaction, which is proportional to the percentage or concentration of the reacted monomer [7]. DTA is a modification of the DSC method and can be conducted to determine the polymerization efficacy of light-cured composite materials. It is simpler to perform relative to the FTIR method, as it does not require special sample preparation in terms of powder generation or sample cutting [24]. Molecular spectroscopy, such as infrared spectroscopy with Fourier transform and micro Raman spectroscopy, is also frequently performed for determining the degree of conversion of composite materials and adhesive systems [25]. This non-invasive method requires minimal sample preparation [21] while permitting quantification of dentin demineralization [25].

The polymerization efficacy of resin-based cementitious materials that contain BisGMA increases with the percentage of TEGDMA in the structure due to the greater mobility and reactivity of TEGDMA molecules [26]. The presence of TEGDMA molecules is also believed to aid the polymerization process, which continues for the next 24 h after light activation [27].

The aim of this study was to evaluate the polymerization efficacy of four dental cement materials characterized by different polymerization mechanisms using FTIR analysis.

## 2. Materials and Methods

In the present study, the FTIR method was adopted to analyze the degree ofpolymerization efficacy of four resin-based dental cement materials, two of which were self-cured and two were dually cured cements (Table 1).

For this purpose, 10 samples of each dental cement were prepared. All samples were prepared in accordance with the manufacturer’s instructions, using circular silicone molds measuring 6 mm in diameter and 2.5 mm in height. Light polymerization was enabled by using the SmartLite PS LED lamp (644.40.010 Dentsplay, York, PA, USA), the positioned unit held in direct contact with the sample for the time duration specified for that particular group.The prepared samples were stored in closed mini tubes in a water bath at 37 °C for 24 h (no light exposure during storage was permitted to avoid any effects on the polymerization mechanism examined during the study).All resin-based dental cements used in our research were stored in hermetically sealed mini tubes (which, according to the manufacturer, prevent any water or liquid penetration) until required for FTIR analysis.

FTIR spectra of the samples were recorded in potassium bromide (KBr) tablets (comprising 0.5 mg sample and 150 mg KBr) in the λ = 4000–400 cm^−1^ spectral range, using the Bomem Hartmann and Braun MB-series FTIR spectrophotometer (Quebec, QC, Canada). All samples were mixed with potassium bromide in the standard way, whereby the required quantities were weighed and then ground in a mill provided for this purpose and then pressed under vacuum, using a standard press comprising the apparatus. The IR spectral analysis was performed 24 h after the polymerization of the cementitious material.

Infrared spectra can be recorded for samples in gaseous, liquid or solid states. Solids are usually tested in the form of a paste or a compressed tablet. The paste is obtained when a finely powdered sample is mixed with a few drops of highly viscous (usually paraffin) oil. This process results in a suspension that can be transformed into a thin film by placing it between two NaCl plates. By mixing the finely powdered sample with a similarly finely powdered KBr, and by compressing this mixture under vacuum, a tablet (or lozenge) is obtained whose IR spectrum reflects the absorption characteristics of the sample. Fourier transform is a mathematical operation that, when applied to an interferogram (using a computer software), directly yields the intensity of the radiation transmitted through the sample as a function of frequency *I*(ν), which corresponds to the IR spectrum recorded on a single-beam instrument.

In order to establish the relationship between the transmittance and frequency (*T*(%) = *I/I_0_* × 100), which is directly obtained when using classical two-beam instruments, the function *I*(ν) is divided by the reference function *I_0_*(ν), which is measured under the same conditions as *I*(ν), albeit in the absence of the sample. In sum, interferogram represents the change in the intensity of monochromatic interference radiation with magnitude X (shift).

In pertinent literature, the degree of conversion (DC) or polymerization efficacy is defined as the percentage of double carbon−carbon (C=C) monomer bonds that transform into single C−C polymer bonds and is calculated as the ratio of double C=C bonds in polymerized and unpolymerized material [16].

The following equation is used to calculate the polymerization efficacy:

DC = [1 − *R_polymerized_*/*R_unpolymerized_*] × 100 (1)

DC = degree of polymerization efficacy or degree of monomer conversion (in %)(2)

R = ratio of peak area at 1638 cm−1 and 1608 cm−1 in polymerized and unpolymerized material(3)

Aliphatic C=C bonds in polymerized and unpolymerized material correspond to the peak at λ = 1638 cm^−1^, whereas aromatic C=C bonds in unpolymerized material correspond to the peak located at 1608 cm^−1^. As aromatic C=C bonds are not subject to change during the polymerization reaction, the peak at 1608 cm^−1^ is adopted as an internal standard when calculating the degree of monomer conversion [17].

For all numerical values, mean, range and standard deviation were calculated. One-factor analysis of variance (ANOVA) was adopted for comparisons among three groups, whereas Student’s *t*-test was utilized for a comparison between two groups, and the potential links between two features were determined via correlation analysis. The SPSS 20 for Windows software was used for statistical data processing, with *p* < 0.05 considered statistically significant. The obtained results are tabulated below.

## 3. Results

The mean values of polymerization efficacy (along with the value range and standard deviation) of the tested materials are presented in Table 2. As can be seen from the tabulated findings, RelyX ARC cement exhibits the lowest polymerization efficacy (61.3%), while that of Variolink II (85.8) and Maxcem Elite is the highest (90.1%).

In Table 3, the mean polymerization efficacy values are separated by the cement type into self-cured and dually cured groups. Although the efficacy of self-cured cements appears to be superior, the difference is not statistically significant (*p* = 0.280), indicating that the polymerization mechanism does not influence polymerization efficacy (Table 4).

As can be seen from Table 5, both Maxcem Elite and Variolink II exhibit a statistically significantly higher polymerization efficacy compared with RelyX ARC and SpeedCEM cements.

FTIR Spectral Analysis

**Figure 2:**
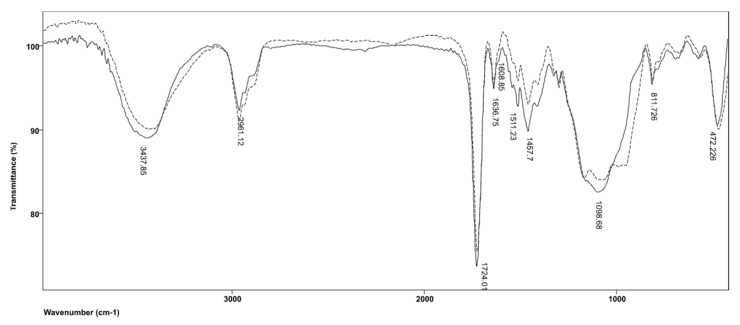
FTIR spectrum produced by the Maxcem Elite resin before and after polymerization.

Figure 2 shows the FTIR spectra of commercial resin-based cement material Maxcem Elite before and after polymerization. It is evident from the graphs that valence C=O vibrations from HEMA are absorbed at λ = 1722 cm^−1^, and, as expected, there is no significant shift in the position of this band in the polymer. Its overtone at 3500 cm^−1^ is obscured by enhanced absorption in the 3700–3200 cm^−1^ range, which originates from the valence absorption of the OH group. As polymer chains include secondary OH groups, as well as intermolecular hydrogen bonds of OH groups, the band related to the absorption of OH groups expands and shifts toward shorter wavelengths, from 3457 cm^−1^ in the monomer to 3437 cm^−1^ in the polymer. Moreover, OH group absorptions in the monomer and polymer are accompanied by deformation vibrations, δOH, which are observed in their spectra at 1456 cm^−1^. The absorptions of symmetric vibrations =CH_2_ groups from the sp^2^ hybridized C atom shift toward lower wavelengths compared to the monomer, from 3141 to 3103 cm^−1^, due to the stronger resonant effect of double bonds in the long polymer chain. Asymmetric valence vibrations, ν_as_(CH_3_), are characterized by the emergence of bands at 2959 cm^−1^ and 2882 cm^−1^.

The characteristic doublet for the ester group located in the HEMA spectrum at 1379 and 1321 cm^−1^ vanishes from the polymer spectrum because, due to polymerization, CH_2_= reaction and formation of aliphatic structure occur, leaving only a band at 1323 cm^−1^ related to CH_3_ deformation vibrations in the δ(CH_3_) plane. The band located at 1298 cm^−1^ originates from the asymmetric valence C−O−C vibrations of HEMA and is also present in the polymer spectrum. In addition, symmetric valence vibrations of the same C−O−C group from methacrylate are absorbed at 1070 cm^−1^ with the accompanying band at 1168 cm^−1^. Owing to the polymerization of C−O−C methacrylate, symmetrical valence vibrations are shifted to 1098 cm^−1^, and the aforementioned band widens because absorption from the C=C−O−C HEMA group occurs at a similar wavelength in the polymer.

**Figure 3:**
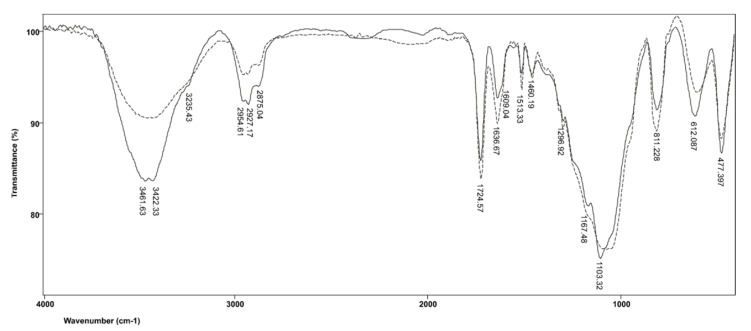
FTIR spectrum produced by the RelyX ARC resin before and after polymerization.

Figure 3 shows the FTIR spectra of commercial resin-based cement material RelyX ARC before and after polymerization. In the spectrum produced by the polymerized resin, the band related to the absorption of OH groups expands and shifts toward shorter wavelengths, from 3446 cm^−1^ in the monomer to 3422 cm^−1^ in the polymer, whereby the band of the OH group in the polymer splits due to the intermolecular hydrogen bonds of OH groups. The OH group absorptions in the monomer and polymer are also accompanied by deformation vibrations, δOH, which appear in their spectra at 1457 and 1460 cm^−1^, respectively. The vibration absorptions of vinyl C=H in the monomers overlap with the vibrations of the free OH groups, while in the polymer, they occur at 3235 cm^−1^, due to the separation of hydrogen-bound and free OH groups. Asymmetric valence vibrations, ν_as_(CH_3_), are characterized by the emergence of bands at 2955 cm^−1^ and 2873 cm^−1^, while the band corresponding to C−H vibrations of the methylene group appears at 2929 cm^−1^. It is also evident that C=O valence vibrations from the ester COO−C group in TEGDMA are absorbed at 1721 cm^−1^ and, as expected, there is no significant shift in the position of this band in the polymer. The presence of aromatic structures is confirmed by bands located at 1612 and 1511 cm^−1^, which are produced by vibrations of the C=C group of the aromatic ring, as well as by absorption at 811 cm^−1^ due to the twisting of =CH-group from disubstituted benzene. The C−O−C asymmetric valence vibrations of TEGDMA occur at 1297 cm^−1^, while the symmetric valence vibrations of the same group in the monomer are absorbed at 1068 cm^−1^. The band at 1168 cm^−1^ corresponding to the valence vibrations of the ester groups in the TEGDMA molecule in the polymer is more pronounced because the bands produced by the absorption of C−O−C groups have shifted. As a result of TEGDMA polymerization, C−O−C symmetric valence vibrations shift to 1103 cm^−1^ because polymerization introduces a larger number of C−O−C groups into the system.

**Figure 4:**
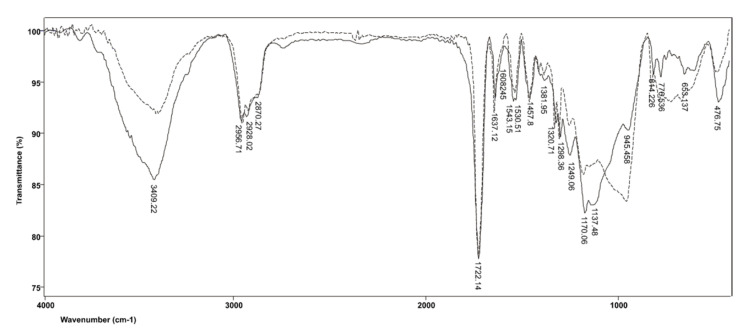
FTIR spectrum produced by the SpeedCEM resin before and after polymerization.

The FTIR spectra produced by commercial resin-based cement material SpeedCEM before and after polymerization are shown in Figure 4. In addition to TEGDMA, SpeedCEM resin also includes UDMA; therefore, the FTIR spectra produced by SpeedCEM differ from those obtained for RelyX ARC precisely in the bands characteristic of UDMA. Consequently, in the FTIR spectrum of the cross-linked resin, the characteristic stretching of urethane bonds (N−H) at 3409 cm^−1^ is evident, as is a combination of urethane carbonyl groups (NH−CO−O) and ester carbonyl bonds (CO−O) at 1722 cm^−1^, along with the band related to C−N bonds, which extends to 1530 cm^−1^. The band located at 1249 cm^−1^ originates from the amide group III (COOC) vibrations. In the polymerized resin, there is a band at 1137 cm^−1^ corresponding to ν(C−O) vibrations, which further amplifies the intensity of symmetric vibrations of the C−O−C group compared with the band at 945 cm^−1^ originating from the deformation vibrations of the C−H group in the aromatic ring. Other bands derived from TEGDMA, discussed in relation to the RelyX ARC resin, are also present in the spectra of unpolymerized and polymerized SpeedCEM resin.

**Figure 5:**
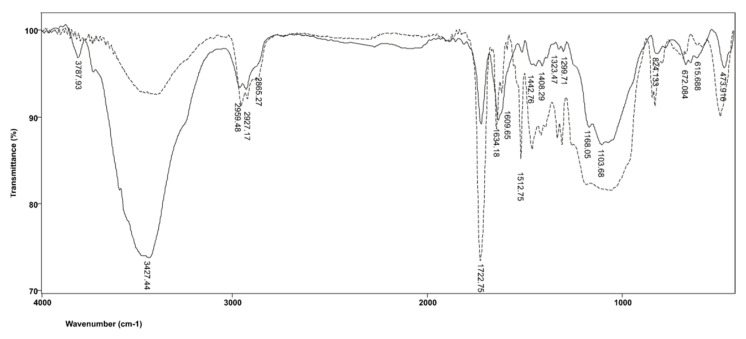
FTIR spectrum produced by the Variolink II resin before and after polymerization.

In addition to TEGDMA and UDMA, the Variolink II resin-based cement also contains BisGMA. Absorption by the ether C−O−C bond from bisphenol A occurs at 1044 cm^−1^, and is slightly shifted relative to the position of the same group in the spectra produced by other materials. The presence of an aromatic pair of substituted benzene rings from bisphenol A is confirmed by a band at 829 cm^−1^, while bands at 1511 and 777 cm^−1^ represent vibrations related to =C−H groups from aromatic structures, as shown in Figure 5.

The FTIR peak assignments for Maxcem Elite, RelyX ARC, SpeedCEM and Variolink II commercial resin-based cement material before and after polymerization are shown in Table 6.

## 4. Discussion

FTIR analysis provides information on the chemical structure of the test material, as well as the degree of polymerization efficacy, which is established by measuring the amount of converted double C=C bonds [7,23]. The FTIR method has been proven to produce reliable results and is thus widely used for determining the degree of conversion from the C=C bond vibrations measured before and after polymerization [10,28]. The advantages of FTIR instruments relative to the classical alternatives are:

- Significantly faster spectrum recording;
- Greater sensitivity;
- Possibility of repeating interferograms;
- Ability to compare the recorded spectra with the spectra previously stored in the computer memory;
- FTIR spectrophotometers cover a much wider spectral range [29].

According to some authors, the main disadvantage of this method is that the analysis results pertain to the polymerization reaction of a part of the sample, which may not be reliable if the sample is not homogenous [24]. In this research, FTIR was employed, as it has been demonstrated to produce reliable findings related to polymerization efficacy of resin-based cement materials.

The degree of polymerization efficacy of resin-based cementitious materials during polymerization is important for the longevity and quality of the restorative procedure, thus determining its long-term clinical success [21,30,31]. An Inadequate polymerization reaction efficacy of resin-based cement materials can undermine their mechanical and adhesive performance [2,32]. A lower degree of polymerization efficacy can result in altered biomechanical properties of the material, in terms of reduced hardness, increased hydrolytic degradation, reduced resistance to fracture and wear as well as significant release of residual monomer, thereby altering material biocompatibility [16,21,33]. Monomer-to-polymer conversion is rarely complete and is generally low in both composite materials and adhesives [19,34,35]. Available empirical data related to resin-based cement materials indicate that monomer-to-polymer conversion rate ranges from 59.3% to 75.0% for self-cured materials, and from 66.6% to 81.4% for dually cured materials [21]. In this study, the mean polymerization efficacy values for the tested materials was within the 61.35–90.07% range. The findings also indicate that the polymerization mechanism does not exert a statistically significant influence on the polymerization efficacy of the studied materials, which was measured at 80.34% and 73.58% for self-cured and dually cured cements, respectively, both of which are satisfactory values. These findings are in accordance with the results of other studies [21].

Still, it is worth noting that the highest polymerization efficacy (90.74%) was obtained for Maxcem Elite, followed by Variolink II (85.81%), SpeedCEM (70.61%) and finally RelyX ARC (61.35%). Moreover, the differences between Maxcem Elite and Variolink II and both SpeedCEM and RelyX ARC were statistically significant. These findings can be attributed to the type of organic resin matrix in the composition of resin-based dental cement [8]. Each of the constituent components of resin-based dental cement materials (organic matrix, filler and coupling agent) affects the mechanical, physical, aesthetic and polymerization properties of the material [13]. Available evidence indicates that the presence of basic methacrylate monomers BisGMA and UDMA results in excellent mechanical properties and low polymerization contraction, but also reduces the monomer-to-polymer conversion rate and the polymerization reaction efficacy. This results in a significant amount of unreacted monomer, which calls into question the biocompatibility of the material. To rectify this issue, other methacrylate monomers such as TEGDMA are added to resin-based cementitious materials, which reduces their viscosity and increases their polymerization efficacy, but also significantly increases the polymerization stress and contraction. The inclusion of TEGDMA into the material structure also increases the filler particle content [9]. As each of the material constituents has some pros and cons, their proportion needs to be balanced. Manufacturers of commercial dental materials consider their exact composition a trade secret (and rarely disclose the type and amount of filler particles), making it difficult to establish the impact of individual components on the polymerization reaction efficacy. However, the Materials Safety Data Sheet does provide the type and approximate percentage of methacrylate monomers, from which their impact on polymerization efficacy of resin-based cement materials can be potentially deduced.The results of Amirouche-Korichi revealed that the polymerization efficacy of dental resin-based composite materials decreases slightly with the increase in opaque filler loadings (La_2_O_3_, BaO, BaSO4, SrO and ZrO_2_ at various volume fractions ranging from 0 to 80 wt.%), but this decrease is not significant [10].Ferrari et al. reported that dental resin-based cements with higher filler content were related to increased polymerization stress, decreased push-out bond strength and increased interfacial nanoleakage [14].

The presence of monomers of high molecular weight, such as BisGMA, enhances the mechanical properties of polymers and reduces polymerization contraction while increasing viscosity. However, as these beneficial characteristics render the clinical application of the material more difficult, low-molecular weight monomers are added into its composition [5]. Thus, in order to reduce the viscosity and achieve a higher filler content, TEGDMA and EGDMA diluents are typically utilized [8,17]. Diluents not only reduce the viscosity of BisGMA but also increase the cross-linking of monomers, resulting in a high degree of polymerization contraction and stress [5,9]. The UDMA molecule in the form of a long linear chain contains one or more urethane groups and two methacrylate groups with molecular weights comparable to BisGMA but with greater flexibility and superior cross-linking capabilities [5,17].

The HEMA fluid molecule has low molecular weight and is capable of sustaining the reaction with unreacted double carbon−carbon bonds (C=C) even after most of the monomer cross-linking process has taken place [12]. The long-chain TEGDMA molecule shows a relatively high degree of polymerization efficacy due to the greater mobility and reactivity of its molecules. Although the degree of monomer conversion to polymers and the consequent polymerization contraction increase with the percentage of TEGDMA in the material, its mechanical characteristics are also compromised [10]. The percentage contribution of TEGDMA monomers in the chemical structure of the material is considered to significantly affect the polymerization reaction. As the quantity of TEGDMA increases, the degree of polymerization efficacy also increases [10].

In the present study, with the exception for Maxcem Elite cement, Variolink II has the highest percentage of TEGDMA, due to which its polymerization efficacy is superior to that of SpeedCEM and RelyX ARC. Maxcem Elite contains only a HEMA monomer in its chemical structure. As previously noted, HEMA is capable of sustaining the reaction with unreacted double C=C bonds even after most of the monomer cross-linking process has been completed [12,36], which is likely responsible for the extremely high degree of polymerization efficacy recorded for the Maxcem Elite cement material. If the resin-based cement contains only UDMA monomers without the inclusion of other monomers as diluents, its conversion efficacy would be reduced [37]. This is evident from the findings obtained for SpeedCEM, which has a slightly higher percentage of UDMA monomers in the composition compared with other tested materials, and thus lower polymerization reaction efficacy. It is worth noting that the degree of conversion values for Maxcem Elite cement reported by other authors are up to three times lower than those obtained in this study, ranging from 14.02 ± 4.95% (self-cured mode) to 26.4 ± 4.19% (dually cured mode) [21]. According to the results of the same study, RelyX ARC exhibited a polymerization efficacy from 11.05 ± 4.16% (self-cure mode) to 37.27 ± 5.01% (dually cured mode) [21].Lopes et al. reported the polymerization efficacy for Variolink (60.5–68.1%) and RelyX (66.3–70.8%), while noting that dually cured resin-based cements show higher polymerization efficacy values compared with self-cured cements, as noted by other authors [38,39]. For Maxcem cement, the polymerization efficacy (%) ranges from 54 ± 3 in the self-cured mode to 61 ± 4 in the dually cured mode [39]. In other studies, however, much lower polymerization efficacy (%) values were obtained for both Maxcem (52.3–59.6) and RelyX (16.5–28.3) [40].

Ultrarapidmonomethacrylates as morpholine carbonyl methacrylate with BisGMA increased polymerization efficacy for 13–21% instead of combination BisGMA/TEGDMA [9].

Some resin-based dental cements require the use of adhesives in order to maximize the adhesive bond strength with the dental structure and indirect restauration (inlay, onlay, crown, bridge and fiber post). In such cases, the tooth or restauration surface is pretreated with the adhesive [7,12]. However, some self-adhesive resin-based cements available on the dental market, such as Maxcem Elite and Speed CEM employed in our study, do not require adhesive use. On the other hand, adhesives are needed for Relyx ARC and Variolink II to maximize the adhesive bond strength. As the application of self-adhesive cements does not require any pre-treatment of dental tissues and the restoration surface, the entire cementation procedure is significantly simplified. Conversely, when working with cements that necessitate the use of adhesives (which is the case for Variolink II and RelyX ARC, as examined in our study), the adhesives employed may not be compatible with the cement, which would consequently weaken the established bonds. For this reason, the Variolink II cement manufacturer recommends the adoption of Excite DSC (IvoclarVivadent AG, Scharn, Liechtenstein, dually cured) or Syntac adhesive (IvoclarVivadent AG, Scharn, Liechtenstein, light-cured). Adper Single Bond Plus adhesive (3M ESPE, Seefeld, Germany) is recommended for use with RelyX ARC as a light-cured adhesive. The information provided in Table 1 is intended as a guidance for dentists based on manufacturers’ recommendations.Thus, each additional step in the preparation of a dental material can contribute to differences in polymerization efficacy, as seen in RelyX ARC, which exhibits the lowest polymerization efficacy.

After light activation, the polymerization process continues for the next 24 h, after which the maximum monomer conversion rate is attained [27], which is why FTIR analysis was performed in this study immediately after this 24 h period had elapsed.

The reported results are attributed to the differences in composition, including amounts of chemicals and photo-initiators, monomeric composition, ratio of diluent monomers and filler content. However, as the exact concentration of each cement component is not provided by the manufacturer, the obtained polymerization efficacy results can only be speculatively linked to the composition.

In most extant studies, polymerization efficacy values were examined in relation to the type of resin-based cement material (self-cured, light-cured or dually cured), while overlooking the impact of the type of ceramic used for restoration [34]. This is a significant shortcoming, given that the clinical success of the restoration procedure depends on the optimal mechanical performance of not only the resin-based cement, but also the ceramic material, whereby resin-based cements are considered to achieve satisfactory mechanical performance if polymerization efficacy (%) is sufficiently high.

Available evidence also indicates that the type and thickness of ceramic material affect the polymerization efficacy of resin-based cements (Variolink dually cured cement). In extant studies, 0.5 mm thick IPS Empress CAD exhibited the highest polymerization efficacy values (%), which ranged from 48.95 to 53.33 for Variolink and from 46.3 to 48.01 for IPS e.max CAD. Moreover, the polymerization efficacy values decreased as the ceramic thickness was increased to 4 mm (IPS Empress CAD 35.23–37.73, IPS e.max CAD 35.03–36.4) [34]. Irradiation length has also been shown to influence the resin-based cement performance, whereby its polymerization efficacy increases with the length of exposure. In our work, the exposure time recommended by the resin-based dental cement material manufacturer [28] was applied.

## 5. Conclusions

The minimum acceptable percentage of resin polymerization has not yet been established, nor is there a definitive recommendation on the choice of cementitious material for use in specific prosthetic restorations. Moreover, when interpreting our findings, it is important to note that the sample of resin-based dental cements analyzed in this study was relatively small, and that their polymerization efficacy was determined using only one method. Nonetheless, our results indicate that polymerization reaction efficacy is not significantly related to the mechanism of polymerization of resin-based cement materials, but is rather influenced by the material type and its chemical structure. Thus, in clinical practice, cementitious materials with a higher proportion of TEGDMA compared with BisGMA are recommended. Clinicians should also consider materials with a higher proportion of HEMA, such as Maxcem Elite, which is a commercial preparation from the group of self-adhesive cements that exhibited the highest degree of polymerization reaction efficacy.While acknowledging the limitations of our study, the findings yielded can still serve as valuable guidelines for future research on resin-based dental cements, which should incorporate additional analyses, such as measurements of unreacted (residual) monomer diffusion.

## Figures and Tables

**Table 1:** Dental cement materials and instruments used in the study.

Material	Manufacturer Lot No.	Polymerization Mechanism	Composition
VARIOLINK II	Ivoclar Vivadent AG, Scharn, Liechenstein LOT M36112 Base LOT M13215 Catalyst	Dually cured cement Requires Excite DSC	BisGMA 10–<20% UDMA 2.5–<10% TEGDMA 2.5–<10% Barium glass Ytterbiumtrifluoride Ba-Al fluorosilicate glass Dibenzoyl peroxide
MAXCEM ELITE	Kerr Bioggio, Switzerland Lot 3485989	Self-cured cement “Self-adhesive”cement	HEMA 19–40% 4 Methoxyphenol Cumene HydroPerOxide Titanium Dioxide Mineral fillers Ytterbium fluoride
SPEEDCEM	Ivoclar Vivadent AG, Scharn, Liechenstein LOT N59834	Self-cured cement “Self-adhesive”cement	UDMA10–25% TEGDMA 10–25% Barium glass Ytterbiumtrifluoride Dibenzoyl peroxide
RELYX ARC	3M ESPE, Seefeld, Germany LOT 3505	Dually cured cement Requires Single Bond Adper	TEGDMA 10–20%TS Copper actetate
SmartLite PS	Dentsplay, York, PA, USA	LED lamp	
Bomem Hartmann and Braun MB-series	Quebec, Canada	FTIR spectrophotometer	

**Table 2:** Minimal, maximal and mean polymerization efficacy, along with standard deviation, for the four studied materials.

Cement	Number of Samples	Minimal Polymerization Efficacy	Maximal Polymerization Efficacy	Mean Value/Standard Deviation
VARIOLINK II	10	0.691	0.987	0.858+/0.900
MAXCEM ELITE	10	0.804	0.987	0.900+/0.067
SPEEDCEM	10	0.378	0.920	0.706+/0.184
RELYX ARC	10	0.306	0.979	0.613+/0.245

**Table 3:** Mean polymerization efficacy values and standard deviations of self-polymerizing and dual-polymerizing cements.

Cement Type	N	Mean Value/Standard Deviation
Self-cured	20	0.803+/0.735
Dually cured	20	0.167+/0.219

**Table 4:** Student’s *t*-test results, indicating the presence or absence of a statistically significant relationship between the polymerization efficacy and the cementitious material type.

	Levene’sTest		*t*-Test				
Polymerization efficiacy	F	Sig.	T	df	Sig.	Mean value	Standard deviation
	2.975	0.093	1.096	38	0.280	0.0676	0.061

**Table 5:** Multiple comparison with polymerization efficacy as the dependent variable.

	Maxcem Elite	Variolink II	Speedcem	Relyx Arc
Maxcem Elite	-	0.043	0.195 *	0.287
Variolink II	-	-	0.152 *	0.245
Speedcem	-	-	-	0.093
Relyx Arc	-	-	-	-

* The *p*-values are below 0.05, indicating statistically significant difference in polymerization efficacy among dental cements.

**Table 6:** FTIR peak assignments for resin-based dental cement materials.

	Frequency, cm^−1^				
Functional Group	Maxcem Elite	RelyX ARC	SpeedCEM	Variolink II	Remark
OH_val_	3457 before polymerization 3437 after polymerization	3446 before polymerization 3437 after polymerization	3396 before polymerization	3424 before polymerization	Valent vibration of OH group
OH_δ_	1456	1460	1457	1442	Deformation vibration of OH group
=CH_2_	3141 before polymerization 3103 after polymerization	3235 after polymerization	3217 after polymerization	/	From C=C group
CH_3_ν_as_	2961 and 2882	2955 and 2873	2956 and 2870	2959 and 2865	
C=C_aromatic_	1608 and 1511	1609 and 1513	1608	1609 and 1512	C=C in aromatic ring
=C-H	811	811	814	814	From aromatic ring
C=O_val_	1724 from HEMA unit	1724 from TEGDMA unit	1722 from urethane group	1722 from urethane group	Carbonyl group
C-O-C	1298 from HEMA unit	1296 from TEGDMA unit	1298 from TEGDMA unit	1299 from TEGDMA unit	Valent asymmetric
C-O-C	1070 and 1168 before polymerization from methacrylic unit	1068 before polymerization from TEGDMA unit	1170 before polymerization from TEGDMA unit	1170 before polymerization from TEGDMA unit	
C-O-C	1098 after polymerization from methacrylic unit	1167 and 1103 after polymerization from TEGDMA unit	1170 and 1137 after polymerization from TEGDMA unit	1168 and 1103 after polymerization from TEGDMA unit	
N-H	/	/	3409 after polymerization	3427 after polymerization	From urethane group
C-N	/	/	1530	1533	From urethane group
N−H in-plane and C−N stretching vibrations	/	/	1249	1247	Amide III
C-O-C	/	/	/	1044	From bisphenol A
C=C	/	/	/	824	From bisphenol A
